# Supplementary material for: Expert-level detection of pathologies from unannotated chest X-ray images via self-supervised learning
Source: Nat Biomed Eng. 2022 Sep 15;6(12):1399–406. doi: 10.1038/s41551-022-00936-9 (PMC9792370; doi:10.1038/s41551-022-00936-9)
Supplement: Supplementary file 1 — Reporting Summary [file 41551_2022_936_MOESM1_ESM.pdf]

nature portfolio

Corresponding author(s): Pranav Rajpurkar

Last updated by author(s): Aug 4, 2022

Reporting Summary

Nature Portfolio wishes to improve the reproducibility of the work that we publish. This form provides structure for consistency and transparency in reporting. For further information on Nature Portfolio policies, see our Editorial Policies and the Editorial Policy Checklist.

Statistics

For all statistical analyses, confirm that the following items are present in the figure legend, table legend, main text, or Methods section.

n/a	Confirmed	
☐	☒	The exact sample size (*n*) for each experimental group/condition, given as a discrete number and unit of measurement
☐	☒	A statement on whether measurements were taken from distinct samples or whether the same sample was measured repeatedly
☒	☐	The statistical test(s) used AND whether they are one- or two-sided<br>*Only common tests should be described solely by name; describe more complex techniques in the Methods section.*
☒	☐	A description of all covariates tested
☒	☐	A description of any assumptions or corrections, such as tests of normality and adjustment for multiple comparisons
☒	☐	A full description of the statistical parameters including central tendency (e.g. means) or other basic estimates (e.g. regression coefficient) AND variation (e.g. standard deviation) or associated estimates of uncertainty (e.g. confidence intervals)
☒	☐	For null hypothesis testing, the test statistic (e.g. *F*, *t*, *r*) with confidence intervals, effect sizes, degrees of freedom and *P* value noted<br>*Give P values as exact values whenever suitable.*
☒	☐	For Bayesian analysis, information on the choice of priors and Markov chain Monte Carlo settings
☒	☐	For hierarchical and complex designs, identification of the appropriate level for tests and full reporting of outcomes
☒	☐	Estimates of effect sizes (e.g. Cohen's *d*, Pearson's *r*), indicating how they were calculated

Our web collection on statistics for biologists contains articles on many of the points above.

Software and code

Policy information about availability of computer code

Data collection	No software was used to collect the data.
Data analysis	The code used to train and evaluate CheXzero is available on GitHub at https://github.com/rajpurkarlab/CheXzero. We developed custom Python code (version 3.6) using the following libraries: Pillow (version 2.2.2, https://pillow.readthedocs.io/en/stable), matplotlib (version 3.3.4, https://matplotlib.org), and numpy (version 1.19.5, https://numpy.org/doc) to visualize and pre-process chest X-ray images.

For manuscripts utilizing custom algorithms or software that are central to the research but not yet described in published literature, software must be made available to editors and reviewers. We strongly encourage code deposition in a community repository (e.g. GitHub). See the Nature Portfolio guidelines for submitting code & software for further information.

Data

Policy information about availability of data

All manuscripts must include a data availability statement. This statement should provide the following information, where applicable:

- Accession codes, unique identifiers, or web links for publicly available datasets
- A description of any restrictions on data availability
- For clinical datasets or third party data, please ensure that the statement adheres to our policy

The main data (CheXpert data) supporting the results of this study are available at https://aimi.stanford.edu/chexpert-chest-x-rays. MIMIC-CXR data are available at

https://physionet.org/content/mimic-cxr/2.0.0 for users with credentialed access. PadChest data are available at https://bimcv.cipf.es/bimcv-projects/padchest. Source data for the figures are provided with this paper.

Human research participants

Policy information about studies involving human research participants and Sex and Gender in Research.

Reporting on sex and gender	The datasets used in this study have sex distributions publicly reported, with the exception of the CheXpert test dataset, which has been hidden for the purposes of official evaluation on the CheXpert leaderboard. We do not report results stratified by sex; however, the design of the study does explore the ability of the method to predict sex from chest X-rays in the PadChest dataset. We report that, for the final labelled PadChest dataset, 80,923 images correspond to women and 79,923 images to men.
Population characteristics	The CheXpert dataset consists of chest radiographic examinations from Stanford Hospital, performed between October 2002 and July 2017 in both inpatient and outpatient centers. Population-level characteristics are unavailable for the CheXpert test dataset, as they are used for official evaluation on the CheXpert leaderboard.<br><br>The MIMIC-CXR and PadChest are datasets available in the public domain.<br><br>MIMIC-CXR is a large dataset involving 65,379 patients imaged at the Beth Israel Deaconess Medical Center Emergency Department during 2011–2016. A total of 377,110 images are available, and are paired with corresponding free-text radiology reports. Each imaging study may contain a frontal and a lateral view.<br><br>The PadChest dataset contains chest X-rays that were interpreted by 18 radiologists at the Hospital Universitario de San Juan, Alicante, Spain, from January 2009 to December 2017. The dataset contains 109,931 image studies and 168,861 images. PadChest also contains 206,222 study reports. The PadChest study reports that the patients' ages range from 0 to 105 years, with a mean of 58.5 years and a median of 62 years. The distribution of the number of images by age is skewed towards older ages, with a long tail for ages under 40. The median birth year of the population was 1953 (birth years ranged from 1904 to 2017), with a standard deviation of 20 years.<br><br>Additional dataset characteristics of the MIMIC-CXR and PadChest datasets are detailed in, respectively, https://www.ncbi.nlm.nih.gov/pmc/articles/PMC6908718 and https://arxiv.org/abs/1901.07441.
Recruitment	No participants were recruited for this retrospective study.
Ethics oversight	The study used data collected retrospectively. Approval of a study protocol was not needed.

Note that full information on the approval of the study protocol must also be provided in the manuscript.

Field-specific reporting

Please select the one below that is the best fit for your research. If you are not sure, read the appropriate sections before making your selection.

☒ Life sciences ☐ Behavioural & social sciences ☐ Ecological, evolutionary & environmental sciences

For a reference copy of the document with all sections, see nature.com/documents/nr-reporting-summary-flat.pdf

Life sciences study design

All studies must disclose on these points even when the disclosure is negative.

Sample size	We used 377,110 chest X-ray images corresponding to 227,835 radiographic studies for model training. For the test set, we used the full CheXpert test dataset, consisting of 500 chest X-ray images labelled for the presence of 14 different conditions. Furthermore, we evaluated the model on 39,053 examples from the PadChest dataset, each of which were annotated by board-certified radiologists. We report results on diagnoses where n > 50.
Data exclusions	No data were excluded.
Replication	The code and data used to train and evaluate CheXzero, which are publicly available (as detailed in the Data-availability and Code-availability statements), can be used to replicate the findings.
Randomization	We didn't require randomization, as no human-subject evaluation was performed.
Blinding	Blinding wasn't relevant to the study, as no human-subject evaluation was performed.

Reporting for specific materials, systems and methods

We require information from authors about some types of materials, experimental systems and methods used in many studies. Here, indicate whether each material, system or method listed is relevant to your study. If you are not sure if a list item applies to your research, read the appropriate section before selecting a response.

Materials & experimental systems

n/a	Involved in the study
☒	☐ Antibodies
☒	☐ Eukaryotic cell lines
☒	☐ Palaeontology and archaeology
☒	☐ Animals and other organisms
☒	☐ Clinical data
☒	☐ Dual use research of concern

Methods

n/a	Involved in the study
☒	☐ ChIP-seq
☒	☐ Flow cytometry
☒	☐ MRI-based neuroimaging
